# Supplementary material for: Causal Relationship between Aging and Anorexia Nervosa: A White-Matter-Microstructure-Mediated Mendelian Randomization Analysis
Source: Biomedicines. 2024 Aug 16;12(8):1874. doi: 10.3390/biomedicines12081874 (PMC11351342; doi:10.3390/biomedicines12081874)
Supplement: Supplementary file 1 [file biomedicines-12-01874-s001.zip › Supplementary Figure/Figure S8/Leaveoneout plot of MR analysis for TL to ubm-b-2053.pdf]

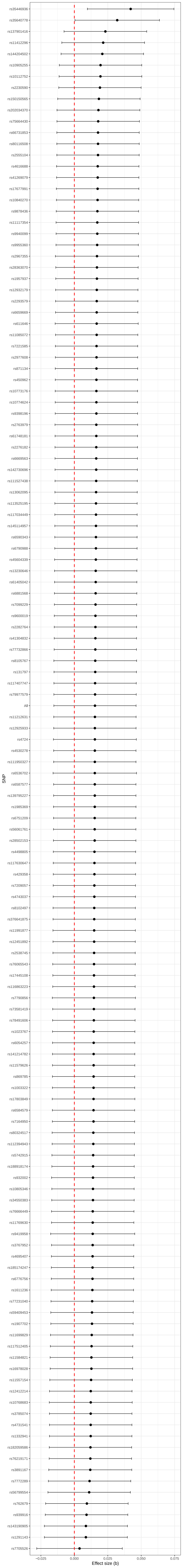
rs35446936
rs35640778
rs137901416
rs11412296
rs144204502
rs10905255
rs10112752
rs2230590
rs150150565
rs202034370
rs75664430
rs66731853
rs80116508
rs2555104
rs4616688
rs41269079
rs17677991
rs10840270
rs9878436
rs11117354
rs9940099
rs9955360
rs2967355
rs28363070
rs1957937
rs12932179
rs2293579
rs6659669
rs611646
rs11085072
rs7221585
rs2977608
rs871134
rs450962
rs10773176
rs10774624
rs9398196
rs2763979
rs61748181
rs2276182
rs6669563
rs142730696
rs111527438
rs13062095
rs113525195
rs117034449
rs145114957
rs6590343
rs6790988
rs45604339
rs13230646
rs61405042
rs6881568
rs7099229
rs9600019
rs2282764
rs41304832
rs77732866
rs8105767
rs131797
rs117407747
rs79977579
All
rs11212631
rs12925933
rs4724
rs4530278
rs111950327
rs6536702
rs6587577
rs139795227
rs1985369
rs6751209
rs56061761
rs28502153
rs4498805
rs117630647
rs429358
rs7209057
rs4743037
rs8102497
rs376641875
rs11991877
rs12451892
rs2538745
rs76065543
rs17445108
rs116863223
rs7790856
rs73581419
rs78491606
rs1023767
rs6054257
rs141214782
rs11579626
rs869785
rs1003322
rs17803849
rs6584579
rs7164950
rs80324517
rs112394943
rs5742915
rs188918174
rs932002
rs10805346
rs34550383
rs76666449
rs11769630
rs9419958
rs3767952
rs4695407
rs185174247
rs6776756
rs1611236
rs77231040
rs59409453
rs1907702
rs11699829
rs117512405
rs11584821
rs16978028
rs11557154
rs12412214
rs10768683
rs3785074
rs4731541
rs1332941
rs182059586
rs76219171
rs3891167
rs7772289
rs56799554
rs762679
rs939916
rs143190905
rs1291143
rs7705526
SNP
-0.025
0.000
0.025
0.050
0.075
Effect size (b)
